# Lipidomics coupled with pathway analysis characterizes serum metabolic changes in response to potassium oxonate induced hyperuricemic rats

**DOI:** 10.1186/s12944-019-1054-z

**Published:** 2019-05-10

**Authors:** Fei Yang, Mingyu Liu, Nankun Qin, Shuangshuang Li, Mengqi Yu, Chengxiang Wang, Qun Ma

**Affiliations:** School of Chinese Materia Medica, Beijing University of Chinese Medicine, Beijing, 102488 China

**Keywords:** Hyperuricemia, Lipidomics, UPLS-Q-TOF/MS, Potassium oxonate

## Abstract

**Background:**

Hyperuricemia as a metabolic disease is usually associated with lipid metabolic disorder. The purpose of this study is to identify potential lipid biomarkers and provide the evidence for the relationship between hyperuricemia and lipid-related diseases.

**Methods:**

Lipidomics-a specialized study of lipid metabolites-has become a highly sensitive and powerful tool for biomarker discovery. In this work, an ultra-performance liquid chromatography-quadruole-time-of-flight tandem mass spectrometry (UPLC-Q-TOF/MS)-based on Lipidomics approach was employed to investigate serum samples from potassium oxonate-treated rats to find potential biomarkers. Principal component analysis (PCA) was used to analyze the MS data to assess the establishment of hyperuricemia model. Orthogonal partial least-squares discriminant analysis (OPLS-DA) in combination with independent samples t-test was performed for biomarker selection and identification.

**Results:**

Thirteen potential biomarkers in rat serum were identified in the screen, and two abnormal metabolism pathways were found, namely glycerolphospholipid metabolism and glycosylphosphatidylinositol-anchored protein biosynthesis.

**Conclusions:**

In this work, the Lipidomics approach based on UPLC-Q-TOF/MS was employed to investigate serum metabolic changes in the rat model, 13 potential biomarkers related to hyperuricemia were identified, primarily involved in glycerolphospholipid metabolism and glycosylphosphatidylinositol-anchored protein biosynthesis. Abnormal glycerophospholipid metabolism pathway may be associated with lipid metabolism disorder caused by hyperuricemia, while the relationship between hyperuricemia and glycosylphosphatidylinositol-anchored protein biosynthesis needs further study.

## Introduction

Hyperuricemia results from overproduction of uric acid and/or reduced excretion of uric acid, it’s generally believed that serum uric acid concentrations > 416 μmol•L^− 1^ or 7.0 mg•d^− 1^ is defined as hyperuricemia [1]. In recent years, with the change of people’s living standard and diet structure, the incidence of hyperuricemia is rising rapidly [2], and tends to be younger. So the prevention and treatment of hyperuricemia become a public health issue of worldwide concern.

Hyperuricemia is not only an important risk factor for the onset of gout [3, 4], but also closely related to metabolic syndrome components [5–7]. Hyperuricemia as a metabolic disease is closely associated with lipid metabolic disorder, so it’s of great significance to explore the pathogenesis of hyperuricemia from the perspective of lipid metabolism.

Metabolomics refers to the comprehensive analysis of endogenous small molecules present in a biological system. The recent technological development of analytical instruments combined with rapid progress in bioinformatics has led to new opportunities to quickly and simultaneously measure and model huge numbers of metabolites in biological samples [8–11]. Lipidomics, an important branch of metabolomics, has highly applied to study characteristics of lipid and to unravel the complex interactions of lipid metabolites [12]. It allows us to better understand pathological process. The UPLC is one of the methodologies used for lipidomics, that can achieve even higher resolutions, higher sensitivities, and rapid separations when compared to conventional LC methods. The UPLC combined with time-of-flight mass spectrometry (TOF-MS), which enables the exact mass measurements, is undoubtedly a suitable system for metabolomics [13].

Potassium oxonate (PO) belongs to triazabenzene compound. Owing to its structure similar to purine ring of uric acid, it can competitively bind with uricase, partly inhibit the activity of uricase, shortly increase the level of SUA in vivo [14]. PO-induced hyperuricemia in mice could serve as an animal model to evaluate the efficacy of drugs [15].

Here, we firstly established hyperuricemic rat model induced by PO. Then, the UPLC-Q-TOF/MS combined with multivariate statistical analysis was applied to generate serum metabolite profiles of rats in control and model groups. MetPA was introduced to analyze metabolic pathways affected by hyperuricemia. The aims of this experiment are to identify possible lipid biomarkers and provide the evidence for the relationship between hyperuricemia and lipid-related diseases.

## Materials and methods

### Instrument

UPLC-Q-TOF/MS was purchased from Waters, USA. ACQUITY UPC CSH C_18_ Column (2.1 × 100 mm, 1.7 μm). Low-temperature and high-speed centrifuge (GL-21 M) was purchased from Shanghai Lu Xiangyi Co., Ltd. ELIASA (Bio-Tek) Blood glucose meter (ONETOUCH Ultra Easy) was purchased from Shenzhen Wei Chuang Li Industrial Co., Ltd. Nitrogen blowing instrument (MTN-2800D) was purchased from Beijing Chengmeng Albert CHAN Technology Co., Ltd. Vortex Mixer (ZH-2BLENDER) was purchased from Haimen City Bellbell Instrument Manufacturing Co., Ltd.

### Reagent

PO (R27A7X13777) was purchased from Shanghai Yuanye Biotechnology Co., Ltd. Uric acid assay kit (20170316), total protein assay kit (20170321), triglyceride assay kit (20170316), and creatinine kit were purchased from Nanjing Jiancheng Bioengineering Institute (China). HPLC-grad solvents (Formic acid, Isopropanol, acetonitrile) were purchased from Fisher (USA). Chloroform and methanol are all analytical regents.

### Preparation of sodium carboxymethyl cellulose solution

0.5 G sodium carboxymethyl cellulose (CMC-Na) was slowly added into 100 ml distilled water. Wait until it’s all swollen up, then shake it until its all dissolved

### Preparation of PO solution

0.6 G PO was dissolved in 0.5% CMC-Na solution (12 ml) to form 5% PO suspension

### Animal model establishment and sample collection

Male SD rats (body weight 200 ± 20 g) were provided by experimental Animal Center of Beijing Wei tonglihua, which were maintained in an environmentally controlled room at 22 °C on a 12 h light/dark cycle and provided with standard diet and water. The rats were randomly divided into model group and control group. The rats in model group (*n* = 8) were intragastrically administered with PO solution by body weight (600 mg•kg^− 1^) while animals in the control group (*n* = 8) given the same volume of CMC-Na solution. Blood sample of each animal was collected from retro-orbital vein at defined periods (7, 14, 21 and 28 days after modeling). After centrifugation for 10 min at 3500 rpm, the supernatant was collected and stored at − 80 °C for reserve.

### Measuring of serum biochemical indicators

The level of Serum uric acid (SUA)、Triglyceride(TG)、Total protein (TP) and Creatinine (CRE) was determined according to the requirements of the kits.

### Serum lipidomics analysis

#### Sample preparation

Serum in 4 °C thawed. 80 μL serum was placed in 1.5 mL eppendorf tube, 320 μL solvent mixture (chloroform/ methanol 3: 1,V/V) was added, mixed in 60 s with eddy current, shaken well, then centrifuged for 10 min with 14,000 rpm, 80 μL organic phase layer (lower chloroform layer) was taken, and dried with nitrogen at room temperature. Then add 100 μL solvent mixture (acetonitrile/isopropanol 1:1, V/V) to dissolve, and centrifuge for 10 min with 13,000 rmp. The supernatant was injected into the UPLC system for analysis.

#### Chromatography and mass spectrometry

The UPLC analysis was performed on ACQUITY UPLC system (Waters). Chromatographic separation was carried out at 45 °C on an ACQUITY CSH C18 analytical column (2.1 × 100 mm, 1.7 μm, Waters, Milford, MA). The mobile phase consisted of 0.1% formic acid (A) and acetonitrile (B), using a gradient elution of 99% A at 0–1 min, 2–99% A at 1–10 min and 99% A at 10–13 min. The flow rate was 0.2 mL•min^− 1^, the temperature of sample manager was set at 4 °C.

Mass spectrometry was performed on a Xevo G2-S Q-TOF/MS (Waters), operating in both negative and positive ion modes. The capillary voltage and cone voltage were set at 3.0 KV and 28 V. The source temperature was set to 100 °C. For positive mode, the cone gas flow was set at 20 L•h^− 1^ and atomized gas temperature was 450 °C. For negative mode, the cone gas flow was set at 50 L•h^− 1^ and atomized gas temperature was 400 °C.

### Data processing

#### Data preprocessing and multivariate statistical analysis

The raw data processed by Markerlynx software to complete noise filter, peak identification, peak matching and normalization were transformed into a two-dimensional data matrix composed of *m/z*, retention time (RT) and peak intensity. Before statistical analysis, the missing value (ion intensity = 0) was processed in accordance with “80% rule” to form a new standardized data array.

SIMCA-P software was used for multivariate statistical analysis and Pareto processing for standardized data matrix. The analysis results were displayed in the form of Scores plots and S-plot diagrams. Metabolites with VIP > 1.0 and *p* < 0.05 were considered meaningful for the model, and then the information of potential biomarkers was searched based on mass-charge ratio and primary and secondary mass spectrum information through Human Metabolome Database (http://www.hmdb.ca) and Lipid Maps (http://www.lipidmaps.org).

#### Network and path analysis

MetPA (http://www.metaboanalyst.ca) is a web-based tool dedicated to the analysis and visualization of metabolomic data within the biological context of metabolic pathways. MetPA combines several advanced pathway enrichment analysis procedures along with the analysis of pathway topological characteristics to help identify the most relevant metabolic pathways involved in a given metabolomic study.

### Statistical analysis

Data were shown as mean ± SD. SPSS 17.0 was used for the statistical analysis. The t-test was used to compare mean between groups, and *P* < 0.05 was considered statistically significant.

## Results

### Serum biochemical indicators analysis

**Fig. 1:**
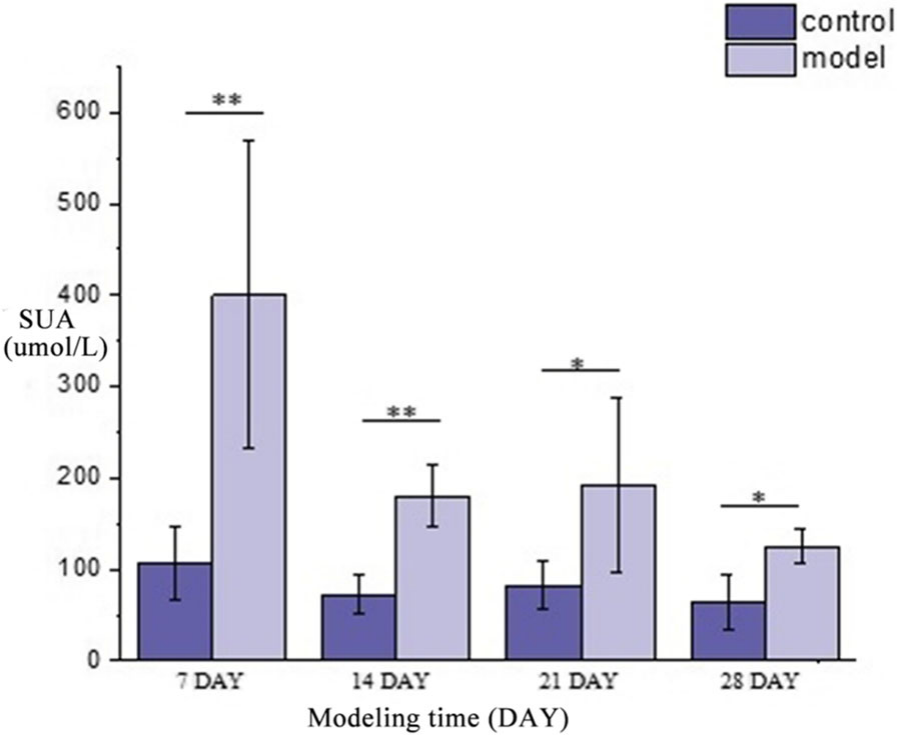
The changing trend and comparison of SUA of rats in model group and control group. * *P* < 0.05. ** *P* < 0.01 Compared with control group

On the seventh day, the SUA of rats in the model group significantly increased (*P* < 0.05) comparing with rats in the control group. As the time went by, the SUA decreased but was still higher than the control group, the result was shown in Fig. 1. However, there were not any obvious differences in TG, CRE and TP of the two groups, as shown in Table 1.

**Table 1:** Comparative result of biochemical indicators at different modeling time (mean ± SD, *n* = 8 rats)

	Group	7 day	14 day	21 day	28 day
SUA (umol•L-1)	Control	106.41 ± 40.38	71.96 ± 21.87	81.90 ± 26.23	63.50 ± 31.06
	Model	400.00 ± 169.00**	179.89 ± 33.52**	192.38 ± 96.21**	125.13 ± 18.93*
TG (mmol•L^− 1^)	Control	0.62 ± 0.40	0.59 ± 0.09	0.6 ± 0.15	1.09 ± 0.72
	Model	0.42 ± 0.14	0.65 ± 0.24	0.51 ± 0.15	1.5 ± 0.31
CRE (umol•L^− 1^)	Control	31.75 ± 17.94	37.47 ± 6.11	51.73 ± 20.49	33.55 ± 17.22
	Model	55.45 ± 53.84	41.04 ± 11.74	35.23 ± 3.11	35.23 ± 30.22
TP (g•L^− 1^)	Control	42.68 ± 5.42	41.08 ± 2.65	46.88 ± 3.84	44.78 ± 3.61
	Model	46.19 ± 5.03	42.34 ± 6.67	49.86 ± 4.58	45.34 ± 4.38

*P < 0.05. **P < 0.01, Compared with control group. *SUA* Serum uric acid, *TG* Triglyceride, *TP* Total protein, *CRE* Creatinine

### Serum metabolism profiling

**Fig. 2:**
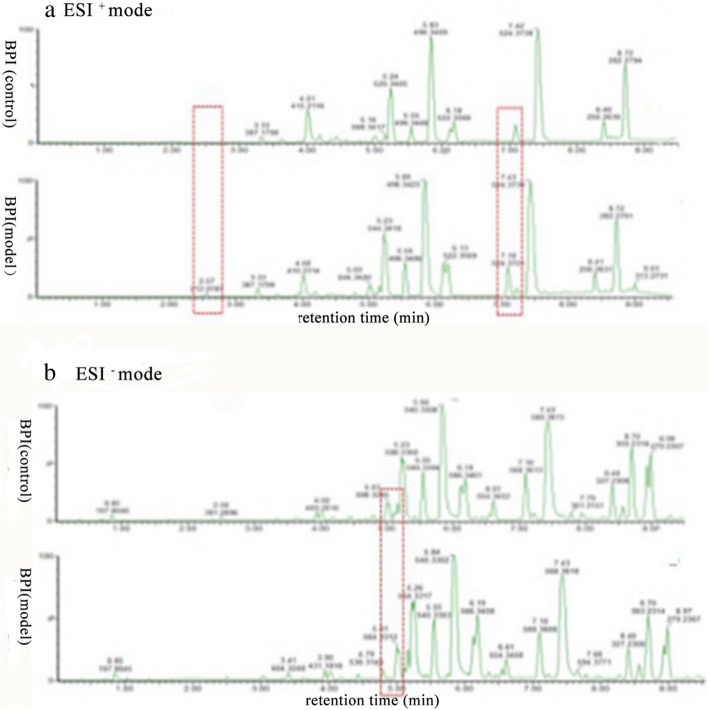
Typical UPLC-Q-TOF/MS BPI chromatograms of serum metabolite profiles from each group in ESI^+^ mode and ESI^−^ mode. **a** BPI chromatograms of the control group and model group in ESI^+^ mode. **b** BPI chromatograms of the control group and model group in ESI^−^ mode

The UPLC-Q-TOF/MS in ESI^+^ and ESI^−^ modes was used to investigate serum metabolic fingerprints of rats in control group and model group. The two base peak intensity (BPI) chromatograms were given in Fig. 2, which could be found that the outlines between two groups were different.

### Multivariate analysis of the serum profiles for model establishment

**Fig. 3:**
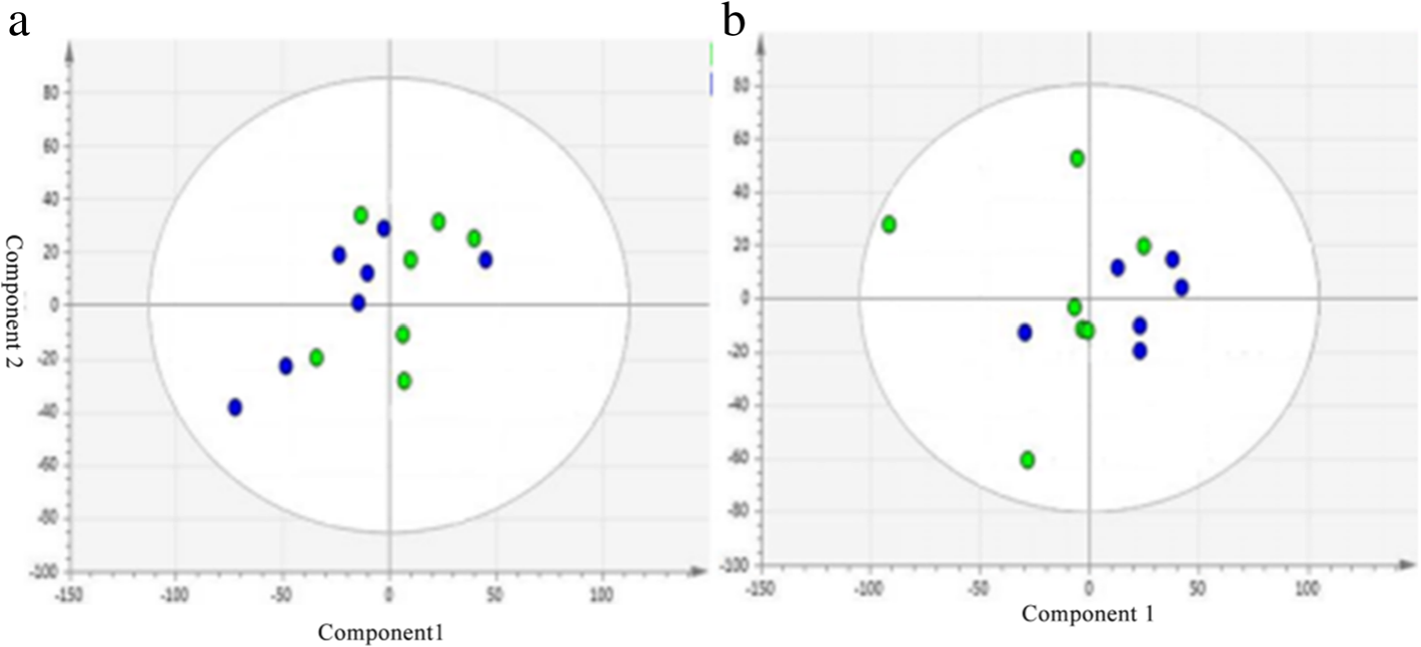
The PCA score plot derived from UPLC-Q-TOF/MS profiles of serum sample from control group and model group in ESI^+^ mode (**a**) and ESI^−^ mode (**b**). C: control group, M: model group

PCA was used to study the pathogenesis of hyperuricemia. Figure 3 shown PCA score plot in ESI^+^ mode and ESI^−^ mode. According to the figure, metabolic pattern of rats behaved differently in different periods. It also revealed that PO would cause disturbance in the metabolic pathway in rats.

**Fig. 4:**
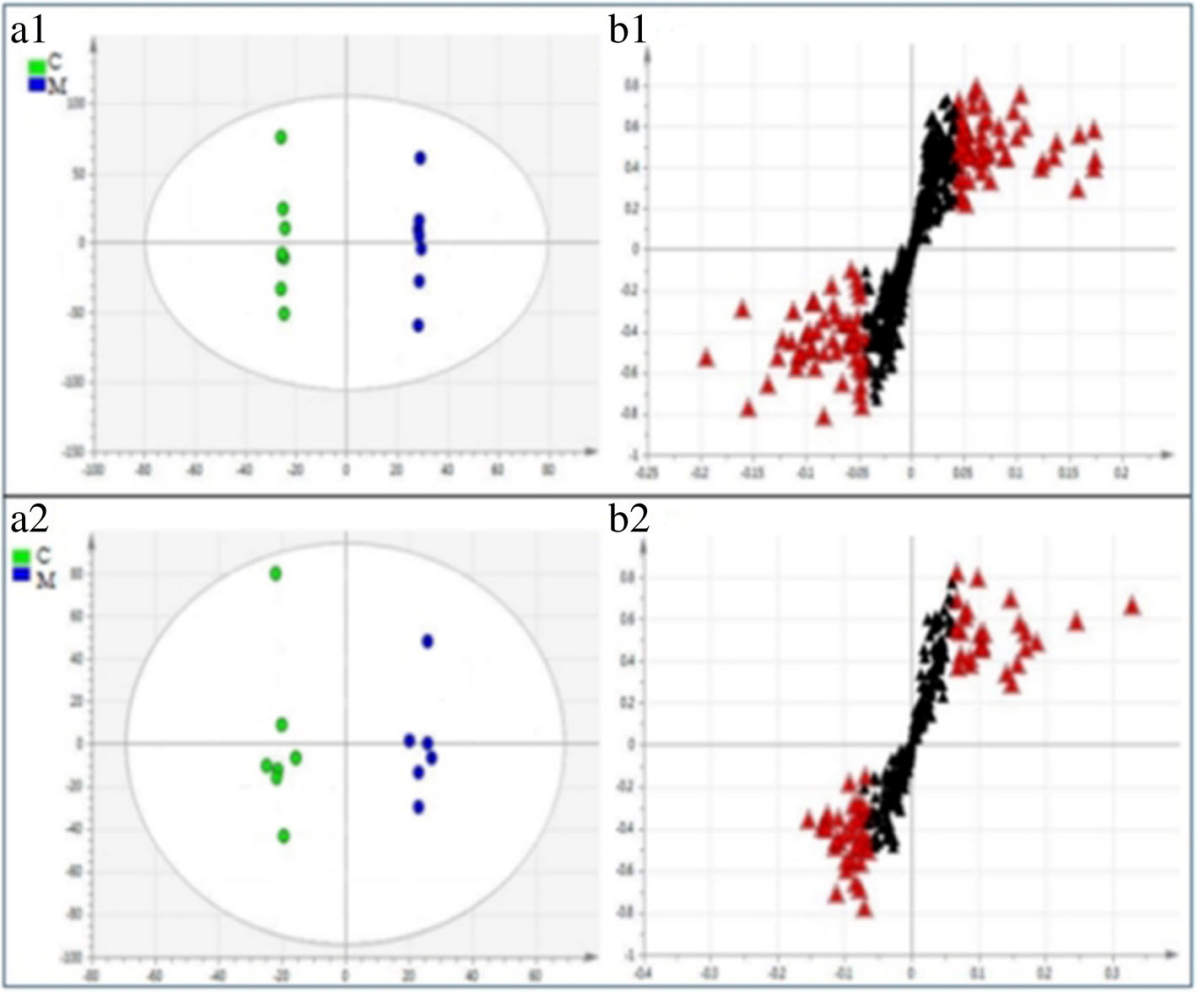
OPLS-DA scores plot and S-plot derived from UPLC-Q-TOF/MS profiles of serum sample from the control group and model group in ESI^+^ mode and ESI^−^ mode. (a1) and (a2) represented the OPLS-DA scores in ESI^+^ mode and ESI^−^ mode, respectively, (b1) and (b2) shown S-plot in ESI^+^ mode and ESI^−^ mode, respectively

OPLS-DA method was utilized to find differential metabolites. OPLS-DA score plot and S-plot of the control group and model group were shown in Fig. 4. In this figure, the parameters of OPLS-DA model in ESI^+^ were as follows: R2 = 1, Q2 = 0.732, and the parameters of OPLS-DA model in ESI^−^ were as follows: R2 = 0.988, Q2 = 0.694. As the figure illustrated, There was a significant separation trend between the two groups, suggesting that the model group had apparent changes compared with the control group in the level of endogenous metabolites. S-plot showed the correlation of each original variable with the first principal component and its importance in the first principal component. Each point in the S-plot represents an original variable. A point farther from the origin is considered with more relation to the first principal component and has a greater contribution rate between groups. The contribution rate of a variable is often described by VIP value. The greater the contribution rate is, the larger VIP value is. In the S-plot, these red triangle dots mean the highest contribution ions with VIP > 1.0, to avoid systematic errors, a variable with a VIP > 1.0 was tested with an independent sample T test.

### Identification of potential biomarkers

**Fig. 5:**
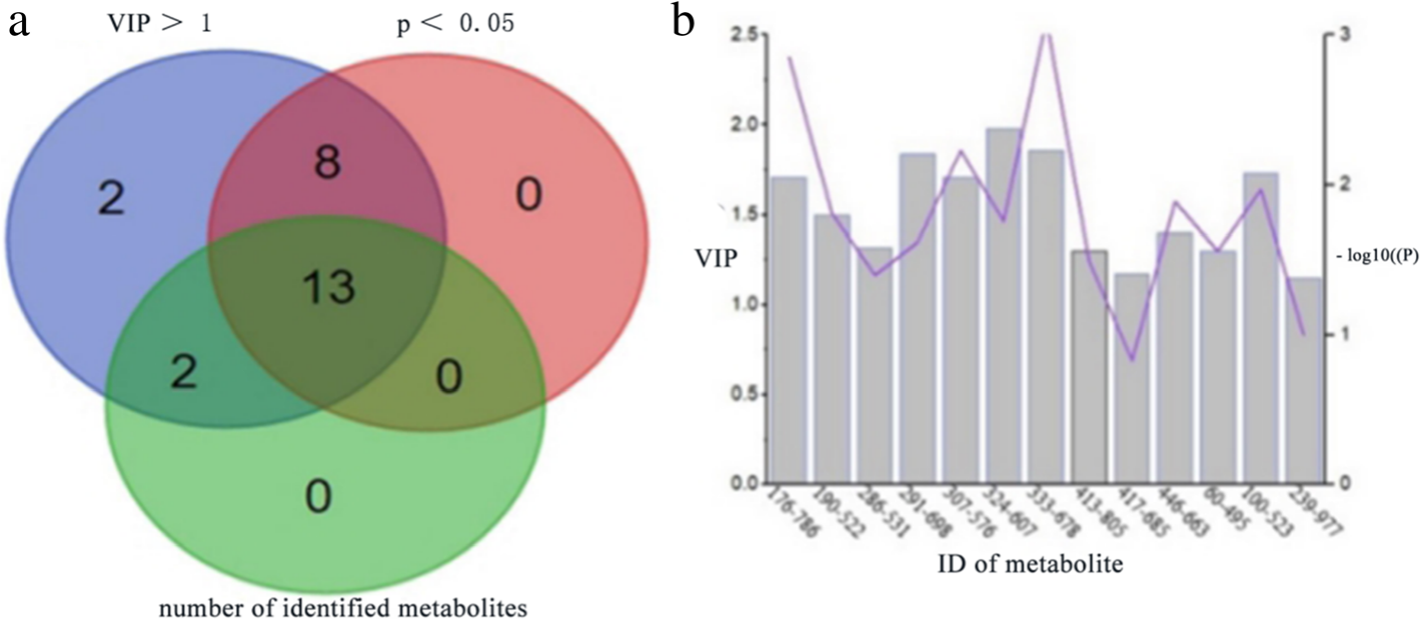
**a** Venn chart displayed the overlapping regions of three sets of elements. 21 differential metabolites with VIP > 1 and *p* < 0.05 were found, 13 variables were identified through searching on network databases. **b** VIP and *p* values of identified metabolites were shown. VIP described the contribution rate of a variable. The greater the contribution rate is, the larger VIP value is. -log10(*p*) represents the difference between groups

In this experiment, a total of 25 variables with VIP > 1 were found, in which 22 variables (*p* < 0.05) could be considered as differential metabolites. These variables were searched on network databases in accordance with their mass-to-charge ratio, then metabolite data and fragmentation pathways obtained from MS/MS analysis were contrasted with ionic properties of public databases to identify their structure and molecular formula, finally 13 variables were identified as shown in Fig. 5a. VIP and *p* values were described in Fig. 5b. Table 2 shown the basic information and the changing trend of identified metabolites.

**Table 2:** Potential biomarkers-related to hyperuricemia. Samples were harvested on 7th day

NO	Inoziation	Rt (min)	*m/z*	Molecular Formula	Compound	Trend
1	ESI-	4.4668	495.6301	C24H50NO7P	LPC(16:0)	↓
2	ESI-	5.0988	523.6832	C26H54NO7P	LPC(0:0/18:0)	↓
3	ESI-	8.9063	978.5961	C65H100O6	TG(18:1/22:6/22:5)	↓
4	ESI+	9.8594	785.5935	C44H84NO8P	PC(18:0/18:2)	↑
5	ESI+	5.8419	521.3481	C26H52NO7P	LPC(18:1)	↑
6	ESI+	8.5336	531.2757	C27H48NO7P	LPE(22:4/0:0)	↓
7	ESI+	7.7872	691.5152	C37H74NO8P	PE(14:0/18:0)	↑
8	ESI+	8.5361	575.7578	C30H58NO7P	LPC(22:2)	↓
9	ESI+	6.7964	607.4577	C32H66NO7P	LPC(24:0)	↑
10	ESI+	7.4282	677.4996	C36H72NO8P	PC(14:0/14:0)	↑
11	ESI+	8.727	806.1031	C46H80NO8P	PC(16:0/22:6)	↓
12	ESI+	7.3395	663.4839	C35H70NO8P	PE(16:0/14:0)	↑
13	ESI+	7.9401	685.4683	C37H68NO8P	PE(14:1/18:2)	↑

Compared with control group. *LPC* lysophosphatidylcholines, *PC* phosphatidylcholine, *TG* triacylglycerols, *LPE* hemolytic cephalin

**Fig. 6:**
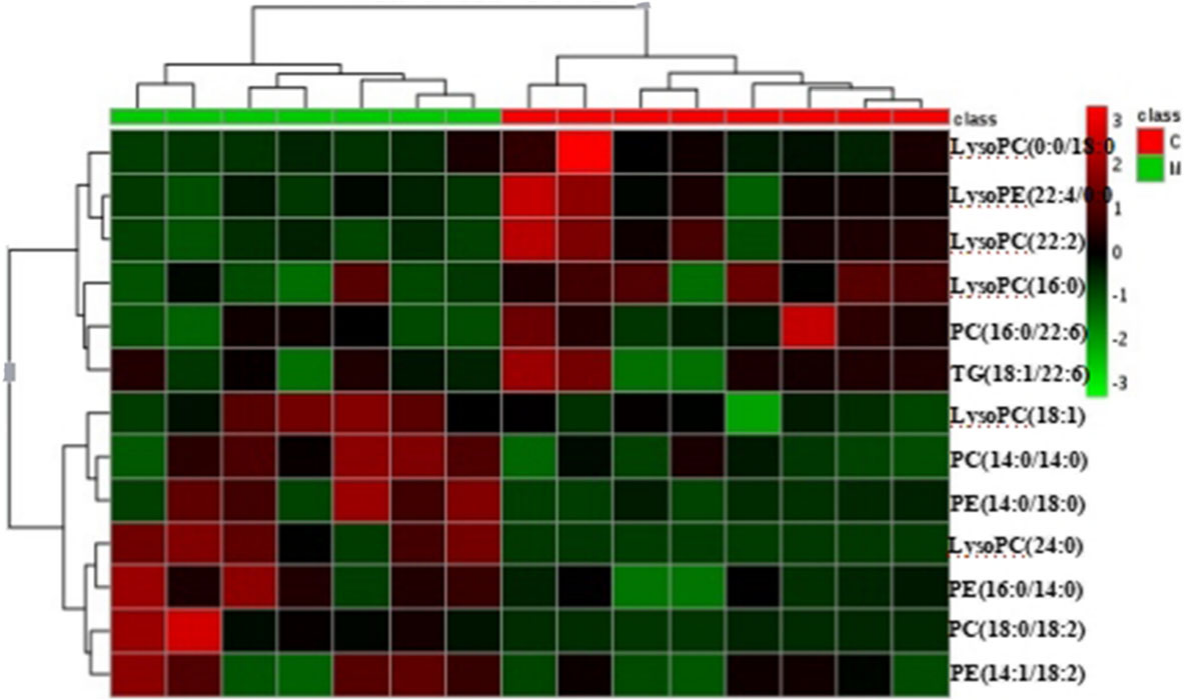
The expression profiles of the 13 lipids in each sample. Hierarchical clustering analysis of the 13 lipids dysregulated between control and model group. For class name, red represents control group and green represents model group. For the expression level of each lipid, red represents high and green represents low

A heat map generated by the result of hierarchical clustering analysis was shown in Fig. 6 to present the expression profiles of the 13 lipids in each sample. The variation trend and fold change of the 13 lipids were shown Table 3, in which saturated fatty acid LPCs were up-regulated in model group, while unsaturated fatty acid LPCs were down-regulated.

**Table 3:** The fold change of 13 lipids

differential metabolites	log2(FC)
LysoPC(24:0)	5.80
PC(18:0/18:2)	3.57
PE(14:0/18:0)	2.68
PC(14:0/14:0)	1.27
PE(16:0/14:0)	1.10
PE(14:1/18:2)	1.06
LysoPC(18:1)	0.66
TG(18:1/22:6)	−0.52
LysoPE(22:4/0:0)	−0.69
PC(16:0/22:6)	−0.82
LysoPC(22:2)	−0.96
LysoPC(16:0)	−1.20
LysoPC(0:0/18:0)	−2.00

**Fig. 7:**
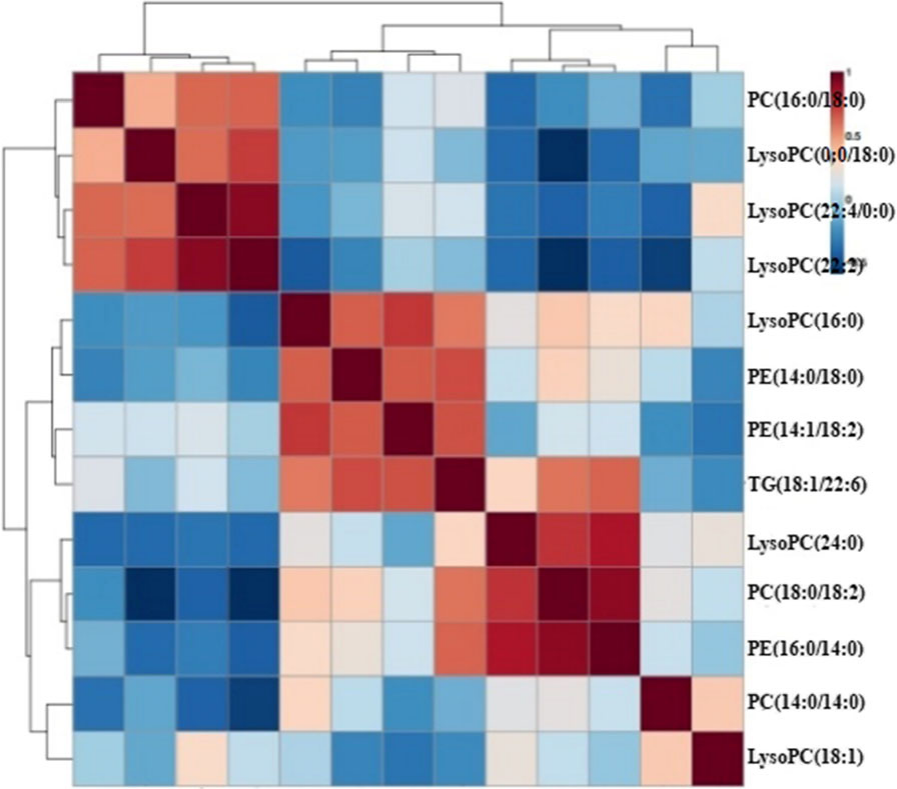
The correlation analysis of biomarkers for hyperuricemia

We also assessed the relationship between 13 biomarkers, as shown in Fig. 7. Different colors can reflect the magnitude of correlation between different metabolites. From overall view, the 13 markers were more closely related, which is of good significance for explaining the pathogenesis of hyperuricemia. Table 4 shown differential metabolites with correlation coefficient > 0.6, including LysoPC(16:0) with PC(16:0/22:6), LysoPC(0:0/18:0) and LysoPC(22:2). LysoPC(18:1) with PC(16:0/22:6) and LysoPC(22:2), and TG(18:1/22:6) with LysoPC(22:2), the analysis of their overall can help to reveal their roles in metabolic pathways.

**Table 4:** Differential metabolites with correlation coefficients > 0.6

differential metabolites	PC(16:0/22:6)	LysoPC(0:0/18:0)	LysoPE(22:4/0:0)	LysoPC(22:2)	PE(14:0/18:0)	PE(14:1/18:2)
LysoPC(16:0)	0.68	0.71		0.99		
PE(16:0/14:0)	0.63					
LysoPC(18:1)	0.97			0.81	0.94	
TG(18:1/22:6)		0.72	0.68	0.75		
PE(14:1/18:2)			0.69			
LysoPC(24:0)						0.76

### Metabolic pathway analysis

**Fig. 8:**
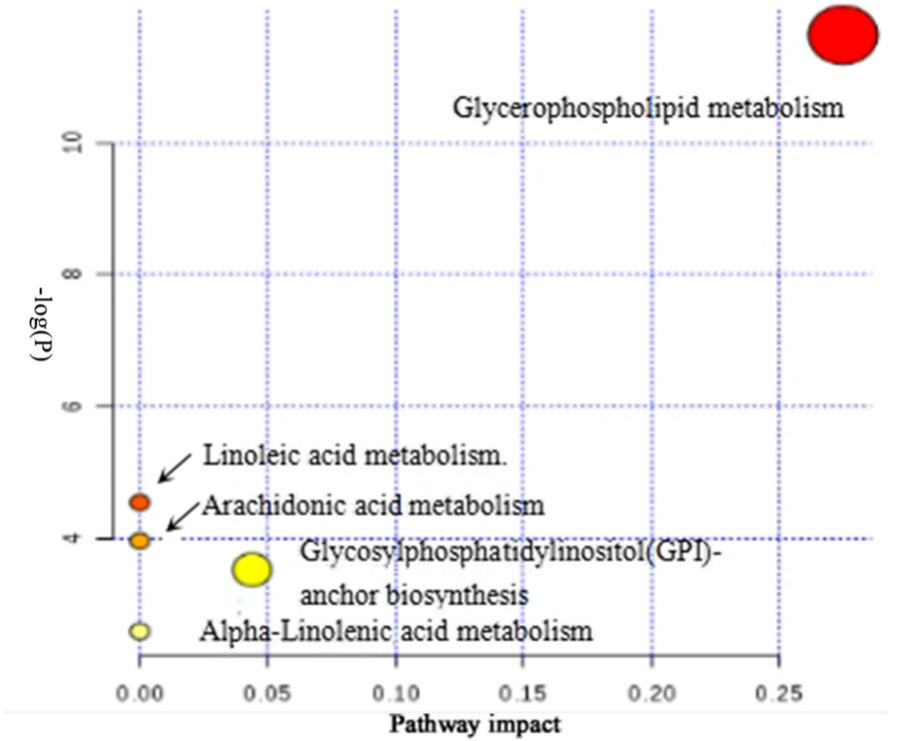
Pathway analysis of the 13 identified metabolites. All matched pathways are plotted according to *p*-value from pathway enrichment analysis and pathway impact score from pathway topology analysis. Colour gradient and circle size indicate the significance of the pathway ranked by *p*-value (yellow: higher *p*-value and red: lower *p*-value) and pathway impact score (the larger the circle the higher the impact score), respectively

The metabolites with obvious changes were imported to MetPA for the metabolic pathway analysis. The result was presented with form of interactive visualization system shown in Fig. 8. Two cardinal metabolic pathways related to hyperuricemia were discovered, namely glycerolphospholipid metabolism and glycosylphosphatidylinositol anchored biosynthesis.

## Discussion

Uric acid is the final metabolit of purine metabolism. The uricase gene of human has been mutated during the process of evolution causing uricase loss, so most metabolits of purine metabolism are excreted in the form of uric acid, consequently human blood uric acid level are much higher than other mammals. But rodents commonly used in experiments have uricase, which can degrade uric acid into allantoin and then excrete [16]. In order to make the animal model more close to human uric acid metabolism, it is crucial to try to eliminate or inhibit the activity of uricase. In this experiment, hyperuricemic rats were induced by uricase inhibitor PO. On the 7th day, SUA reached its peak. With the prolonging of time, the SUA decreased. The reason for this may be that high level of uric acid stimulates uricase to increase its activity and accelerates conversion of uric acid to allantoin leading to decrease in uric acid level. It also proved that PO-induced hyperuricemia seemed to be acute and transient models which is consistent with a previous study [17], despite that acute model gives us the first information on their effectiveness in vivo. This may be why PO is often used together with other molding agents.

**Fig. 9:**
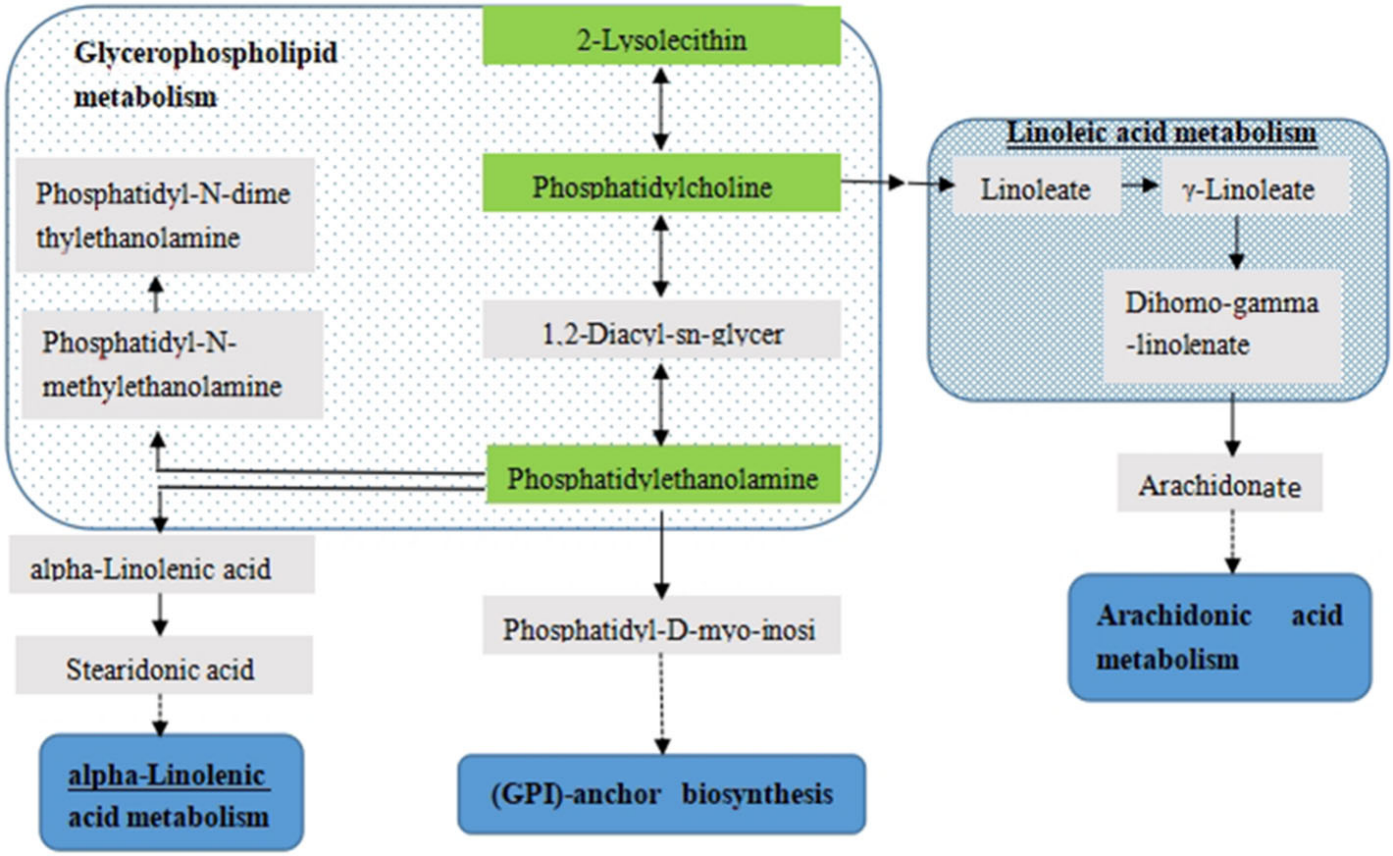
The metabolic network profile

In this study, 13 differential metabolites were identified that are highly associated with the metabolic changes resulting from PO-induced hyperuricemia. To explore these molecular potential functions of serum metabolite biomarkers, metabolic pathway analysis was performed by searching the MetPA database. We found that these metabolites were primarily involved in glycerophospholipids metabolism and glycosylphosphatidylinositol-anchored protein biosynthesis. By correlating the metabolic pathways, a metabolic network was constructed shown in Fig. 9. The disturbed metabolic pathways are discussed in detail below.

Glycerophosphatide is one of the most abundant phospholipids in the body. It is also one of the components of bile and membrane surfactant besides biofilm. In this study, glycerophosphatides of 16-C and 18-C fatty acid chains including PC(18:0/18:2)、PE(14:0/18:0)、PE(16:0/14:0)、LPC(18:1) were obviously increased in the serum of the hyperuricemia model group relative to the control group. Under normal physiological conditions, the amount of these lipid metabolites is not large, but under inflammation, they can aggregate and produce obvious pathological features. Hyperuricemia can induce inflammation by promoting proliferation and inflammation, which may cause these lipis to be increased with hyperuricemia.

PC and LPC were mainly involved in glycerophospholipids metabolism. In this study, glycerophospholipid metabolism is implicated in hyperuricemic rats. Consistent with these findings, previous study about plasma metabolism differences between hyperuricemia patients and healthy individuals has found that abnormal lipid metabolism is one of the metabolic characteristics associated with hyperuricemia [18]. Glycerophosphatidylcholine metabolism has a certain metabolic network, and the disorder of the network may bring many diseases. The result reflects that hyperuricemia is closely related to many diseases.

Glycosylphosphatidyl inositol (GPI), protein-monosaccharide-fatty acid compounds, is one of the approaches for proteins binding to cell membranes. Glycosylphosphatidylinositol-specific phospholipase D (GPI-PLD) is the only phospholipase that can hydrolyze GPI-anchored proteins, selectively releasing anchoring proteins under specific conditions, simultaneously regulating GPI anchor synthesis, and ultimatly controling GPI-anchored protein expression [19]. GPI-anchored proteins, like CD24、CD87, can adjust the adhesion and migration of leukocytes [20], while the invasion and adhesion of leukocytes to endothelial cells is one of the key early events of atherosclerosis. It has been found that CD16, T-CAD, CD87, CR-1 and other GPI-anchored proteins are closely related to the occurrence and development of atherosclerosis. Because PO-induced hyperuricemia is associated with the kidney inflammation in mice [21], the inflammatory response system can induce or inhibit the release and synthesis of GPI-PLD. In this study, the metabolic pathway of glycosylphosphatidylinositol-anchored protein biosynthesis was affected, which indicated PO-induced hyperuricemia might have an impact on expression of GPI-PLD resulting in disorders of GPI-anchored proteolysis and synthesis.

## Conclusions

In this work, the Lipidomics approach based on UPLC-Q-TOF/MS was employed to investigate serum metabolic changes in the rat model, 13 potential biomarkers related to hyperuricemia were identified, primarily involved in glycerolphospholipid metabolism and glycosylphosphatidylinositol-anchored protein biosynthesis. Abnormal glycerophospholipid metabolism pathway may be associated with lipid metabolism disorder caused by hyperuricemia, while the relationship between hyperuricemia and glycosylphosphatidylinositol-anchored protein biosynthesis needs further study.

## Abbreviations

PCA: Principal component analysis
PLS-DA: Partial least squares-discriminate analysis
PO: Potassium oxonate
